# An investigation of temporal resolution parameters in cine‐mode four‐dimensional computed tomography acquisition

**DOI:** 10.1120/jacmp.v9i4.2819

**Published:** 2008-10-29

**Authors:** Yildirim D. Mutaf, Debra H. Brinkmann

**Affiliations:** ^1^ Division of Medical Physics, Department of Radiation Oncology Mayo Clinic Rochester Minnesota U.S.A.

**Keywords:** 4DCT, temporal parameters, image quality, respiration motion

## Abstract

The accuracy of four‐dimensional computed tomography (4DCT) imaging depends on temporal characteristics of the acquisition protocol—for example, the temporal spacing of the reconstructed images (also known as cine duration between images) and the gantry rotation speed. These parameters affect the temporal resolution of 4DCT images, and a single default acquisition protocol, as commonly used in most clinics, may be suboptimal for a subset of respiratory motion characteristics. It could lead to substantial inaccuracies in target delineation. The aim of the present study was to evaluate the interplay between parameters affecting temporal resolution and the accuracy of the resulting images.

We acquired 4DCT images of cylindrical phantoms under repetitive motion induced by a translation platform. Acquisition settings varied with respect to temporal spacing, gantry rotation speed, and motion period of the phantoms. Reconstructed images were sorted into 10 phase bins and were compared to static phantom images acquired at corresponding positions of the respiration phase. Acquisitions with different temporal spacing did not play a significant role in the amount of motion observed in full‐cycle maximum intensity projection images. Target delineation accuracy at end‐of‐inhalation phase was observed to be constant up to a threshold in the value of the reconstruction interval, beyond which it varied arbitrarily. This threshold was found to be correlated with the number of phase bins and the motion period. No observable variations were noted with images from the end of exhalation when temporal spacing was varied. Target delineation accuracy was observed to be enhanced in acquisitions using faster gantry rotation speeds. An evaluation of the acquisition parameters needs to be performed depending on the period of the motion and limiting factors such as the availability of acquisition settings, X‐ray tube workload, image storage, and processing power.

PACS numbers: 87.53.Xd, 87.57.‐s, 87.57.Gg, 87.59.Fm

## I. INTRODUCTION

Intrafraction respiration motion has been shown to introduce significant uncertainties into the visualization and localization of anatomy.^(1)^ Additionally, lack of temporal information for tumor volumes or nearby healthy critical structures may cause dosimetric errors and therefore lead to suboptimal radiation treatments.^(^ ^2^ ^–^ ^4^ ^)^ With the advent of new technologies incorporating respiration motion into existing imaging modalities—for example, four‐dimensional computed tomography (4DCT)^(^ ^5^ ^–^ ^9^ ^)^—the ability to identify the full excursion of critical anatomy improved substantially. Additional temporal information provided with 4DCT imaging has been shown to facilitate the application of motion‐incorporated radiotherapy techniques,^(4)^ providing increased conformality and therapeutic advantage in the treatment of mobile tumors.

Despite simplicity of operation in principle, 4DCT imaging systems are also prone to errors that could be propagated to undesirable dosimetric consequences. Potential sources for such errors include irregular breathing by patients,^(10)^ inaccurate assessment of respiratory motion,^(11)^ ^,^ ^(12)^ or inappropriate selection of acquisition parameters. The focus of the present study was on selection of the 4DCT acquisition parameters and subsequent evaluation of how the selected temporal parameters affect the accuracy of the final images. Conventional X‐ray imaging parameters such as tube current, peak voltage, collimation size, and slice thickness were kept consistent while acquisition parameters that affect the temporal resolution were investigated:

- Temporal spacing in a cine scan (Δ*t*)
  The Δ*t* parameter controls the temporal spacing between two consecutive images reconstructed at the same scan position. It is also called “cine time between images” in some commercial scanning systems.
- Gantry rotation speed $\left(T_{R}\right)$

The $T_{R}$ parameter represents the time it takes for the X‐ray tube to make one full rotation. It equals the length of the projection data used for the reconstruction of a single image under full segment reconstruction mode.

**Figure 1:**
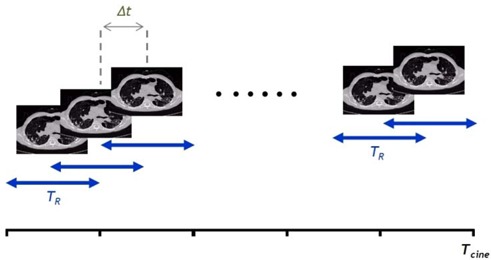
The four‐dimensional computed tomography temporal acquisition parameters: temporal spacing (Δ*t*) and gantry rotation period $\left(T_{R}\right)$.

Fig. 1 illustrates these parameters and their roles in 4DCT image reconstruction.

## II. MATERIALS AND METHODS

We analyzed images from more than 50 4DCT studies acquired using a 16‐slice GE LightSpeed RT scanner (GE Healthcare Technologies, Waukesha, WI). The image studies were acquired under cine mode, a modified axial scanning mode that enables multiple gantry rotations for each couch location. Images were acquired with slice thicknesses of 1.25 mm and a collimation thickness of 10.0 mm. Apeak X‐ray tube voltage of 120 kV and a tube current of 200 mAwere selected for scanning, with a $T_{R}$ of 1 s (gantry rotation). For shorter gantry rotation periods (<1 s), we adjusted the milliamperes such that the total milliampere‐seconds $\left(\text{mA s}:\;\text{mA}\times T_{R}\right)$ was kept constant so as to establish comparable noise levels in reconstructed images.

**Figure 2:**
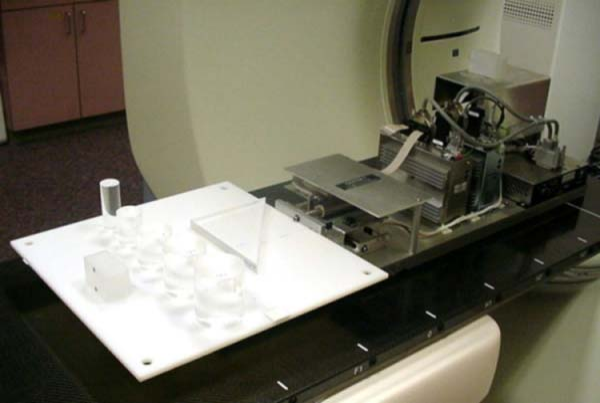
The motion platform used in this study. Cylindrical phantoms were placed on the platform, together with the Varian Real‐Time Position Management system marker block, and moved periodically along the longitudinal axis of the phantom (in and out the scanner bore).

A motion platform was used to simulate respiratory motion for phantoms placed atop the platform. The platform consists of three linear motors (LinMot, Delavan, WI), two moving along the horizontal axes orthogonal to each other, and one moving along the vertical axis as shown in Fig. 2. A three‐dimensional controller and an encoder system executes position commands for the platform at frequencies of up to 500 Hz. With the use of custom‐developed control software, the platform can simulate functional forms of respiration motion at a measured accuracy of 0.0±0.3mm.^(11)^

Motion management is performed retrospectively using the Varian Real‐Time Position Management system, ver. 1.6.5 (Varian Medical Systems, Palo Alto, CA). An infrared camera recording the motion of a surrogate marker block placed atop the motion platform tracks the simulated respiratory motion.

For quantitative analysis of target delineation accuracy, acrylic cylindrical phantoms are placed on the motion platform such that the motions of the surrogate marker block traced by the respiratory management system and the phantoms are always identical. Five cylinders of radii 1.43 cm, 1.91 cm, 2.54 cm, 2.86 cm, and 3.18 cm (here called phantoms 1 – 5, in order of increasing radius) were placed as in the setup shown in Fig. 2, modeling target objects observed clinically. The phantoms were arranged such that a coronal view of captured images shows the circular cross‐sections of phantoms. In the acquired cine scans, phantoms were mobile along the longitudinal direction of the scanner, perpendicular to the scan plane, following the periodic motion of equation 1,^(13)^

(1) $$z(t)=z_{0}+Z\cdot \cos^{6}(2\pi t/T),$$

where the constant parameter Zrepresents the maximum excursion of the motion observed during a single period *T*. Throughout the study, we used a peak‐to‐peak motion amplitude of 2.0 cm *(Z)*.

We acquired 4DCT scans with a gantry rotation period ($T_{R}$ of 1 s while the phantoms were under simulated respiratory motion with periods (7) of 3 s, 4 s, and 5 s. These images were then reconstructed offline with temporal spacing *(At)* of 0.15 s, 0.20s, 0.25 s, 0.30s, 0.35 s, 0.40 s, 0.45 s, 0.50 s, 0.60 s, 0.70 s, 0.80 s, 0.90 s, and 1.00 s, corresponding to 39 different 4DCT studies. To study the role of various gantry rotation speeds, 4DCT studies corresponding to faster gantry scans $\left(T_{R}=0.5\;s,0.6\;s,0.7\;s,0.8\;s,\;\text{and}\;0.9\;s\right)$ were acquired and reconstructed with a temporal spacing of 0.2 s.

Reconstructed images were then sorted into 10 phase bins for final evaluation using GE Advantage 4D software (GE Healthcare Technologies). Each phase bin is therefore a three‐dimensional image constructed from axial slices with similar phases. These three‐dimensional images were then compared to static scans, which were acquired in axial mode while the programmable platform was positioned at longitudinal locations corresponding to 0%, 10%, 20%, 30%, 40%, and 50% phases of the motion. Maximum intensity pixel (MIP) images were constructed from the phase images and were used to assess the effect of acquisition parameters on a wider time range, such as the full motion period.

Compared to static scans, scans using the acquisition parameters studied here show effects that reflect differences in residual motion, motion artifacts, and other motion blurring that are finally quantified in terms of the delineated size of the phantoms. Acrylic phantoms have Hounsfield units in the reconstructed CT images close to those of muscle tissue (40 HU) and are efficiently visualized using a pulmonary window and level (1000 HU and −700 HU). A coronal view of the images showing the circular cross‐section of the phantoms was extracted as an 8‐bit grayscale image with a resolution of 512×512 pixels for further analysis. These 8‐bit images were then imported into the ImageJ image processing software (National Institutes of Health, Bethesda, MD), and measurements with respect to the relative sizes of the phantoms were performed using the automatic contouring and other image processing tools available in ImageJ.

We investigated the temporal spacing parameter (Δ*t*) in terms of its effect on target delineation for the full range of motion and for individual phase images (0% peak inhalation and 50% peak exhalation). For the full range of motion, we compared MIP images constructed from all phase images of the phantoms under simulated motion to static phantom images.

We investigated the effects of gantry rotation speed by comparing MIP images involving a range of motion under various gantry rotation speeds for a fixed temporal spacing value of 0.2 seconds. Maximum intensity pixel images from the full respiration cycle (that is, [0% – 90%] phase window) and those that were constructed from several different phase bin ranges were centered at the end of exhalation for quantitative comparisons—that is, [40%, 60%], [30%, 70%], and [20%, 80%]. The motivation for the selection of shorter phase windows was based on scenarios in which respiratory gating could be performed at the end of exhalation. Therefore selection of the phase windows investigated in the study corresponds to gated treatment duty cycles of 3.3, 2.0, and 1.4 respectively.

## III. RESULTS AND DISCUSSION

### A. Temporal spacing (Δ*t*)

**Figure 3:**
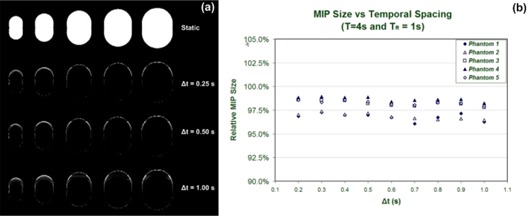
Maximum‐intensity pixel (MIP) images for the full range of motion are constructed using static images and images of phantoms under motion. (a) To highlight differences and to facilitate quantitative comparisons with static counterparts, only subtraction images for the phantoms are shown, corresponding to a selection of temporal spacing parameters. (b) Apparent sizes of the phantom objects in MIP images are shown relative to the corresponding static images.

Fig. 3(a) shows MIP images of static phantoms created offline and pixel subtraction images for MIP images of the phantoms under motion acquired with various temporal spacing values (Δ*t*). The MIP images of static phantoms were created by applying a pixel maximum value operator to images of static phantoms.

Fig. 3(b) plots the relative size of each phantom in the motion MIP images with respect to those in the static images. For the full range of motion represented by the MIP images, the phantom size did not present any considerable dependence on the selection of temporal spacing between images (Δ*t*). A slight underestimation of size is apparent in the 4DCT MIP images for all phantom sizes. This underestimation is attributable to the fact that the MIP construction from static images is created using phase images, including the exact 0% (peak inhalation) and 50% (peak exhalation) phases—that is, the motion extremes. However the images representing the phases at the motion extremes for the 4DCT MIP images may not fall at the actual motion extreme, because they are binned from a range of possible actual phases. For example, the 0% phase images contain axial images with calculated phases ranging from 96% to 3%.

**Figure 4:**
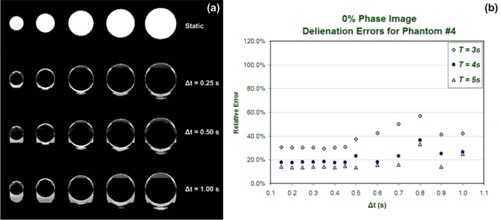
(a) End‐inhalation images (0% motion phase) are shown from static and subtraction images acquired with a selection of temporal spacing parameters. (b) Relative delineation errors as defined in the text are shown for one phantom as a function of the temporal spacing parameter.

To represent situations in which a subset of motion is considered, we also evaluated the effect of temporal spacing on individual phase images. Comparisons of single phases were used the 0% and 50% phase images corresponding to images at the end of inhalation and the end of exhalation respectively. Fig. 4(a) shows subtraction images between the 4DCT and static images from the end of inhalation phase (0%), in which the intensity values represent the absolute value of differences between corresponding pixels of the two images. Errors in target definition were measured from the subtraction images and reported as the ratio of non‐zero pixels within a region of interest created around the phantom to the non‐zero pixels in the static image. The only non‐zero pixels in the static image were a result of the presence of phantom objects, and therefore the pixel count within a specific region of interest in static images is a measure of the actual cross‐sectional size of each phantom. Pixel differences were normalized with respect to the number of pixels in the static image and were plotted in Fig. 4(b) for phantom 4 with a radius of 2.86 cm.

**Figure 5:**
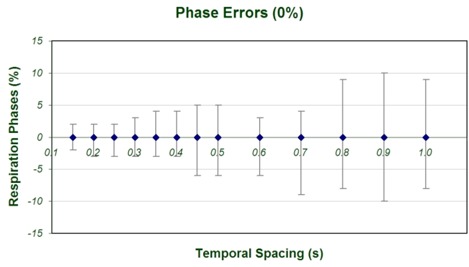
Maximum phase errors within the construction of the 0% phase bin $\left(T=4\;s,\;T_{R}=1\;s\right)$ are shown as a function of temporal spacing, Δ*t*.

Below a particular threshold value, the delineation errors show no dependence on Δ*t*. However, deviations with respect to the static images fluctuated arbitrarily beyond this threshold value, which appeared to be about Δt=0.5s. We observed that this threshold was consistent for respiration motion periods of 3 s, 4 s, and 5 s; however, the magnitude of the relative errors was observed to be greater for shorter motion periods. This threshold corresponds to the minimum statistical sampling necessary for defining an accurate flow of motion given the phase binning chosen in the analysis. Under‐sampling is evidenced by an amplified range of phase errors for each phase bin as temporal spacing increases. The range of maximum phase errors for the 0% phase bin $\left(T=4\;s,T_{R}=1s\right)$ is shown in Fig. 5 as a function of Δ*t*. These errors are calculated by comparing the calculated respiration phase of individual slice images and the value of the phase bin to which the relevant image is assigned. For a temporal spacing parameter of 0.45 s and larger, all images contributing to the 0% phase image had phase errors exceeding ±5%. The magnitude of the spatial artifacts resulting from these phase errors varied with the respiration period, because the amount of residual motion changed, but the reconstruction interval time (or gantry rotation speed) was unaltered.

**Figure 6:**
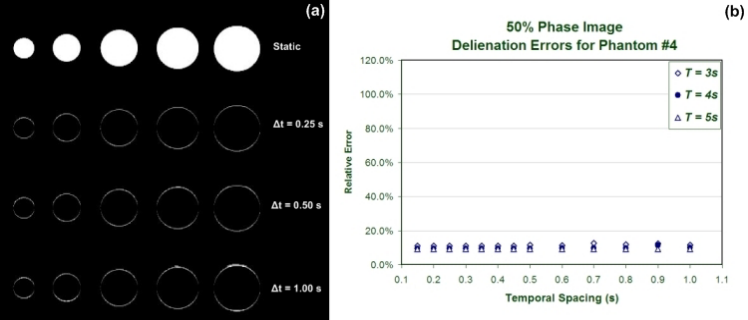
(a) End‐of‐exhalation images (50% motion phase) are shown from static and subtraction images acquired with a selection of temporal spacing parameters. (b) Relative delineation errors as defined in the text are shown for one of the phantoms as a function of temporal spacing.

Phase images corresponding to the end of exhalation (50% phase) did not present any dependence on the temporal spacing between images. Fig. 6 shows subtraction images for the 50% phase and the plot of relative target delineations with respect to the static images. Despite the fact that the 50% phase‐bin images were subject to sampling problems as Δ*t* increased, the resulting phase errors did not produce the same target delineation errors as the 0% phase did, because of the amplitude plateau at this phase location.

### B. Gantry rotation speed (TR)

**Figure 7:**
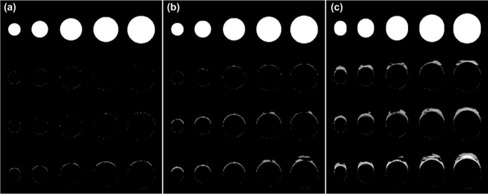
Effects of gantry rotation speed for several phase window selections in the construction of maximum‐intensity pixel (MIP) images. The first row of images is obtained from the corresponding phases of the static images. The remaining rows present MIP images for gantry rotation speeds of 0.6 s, 0.8 s, and 1.0 s, from top to bottom respectively. (a) [40%, 60%]. (b) [30%, 70%]. (c) [20%, 80%].

**Figure 8:**
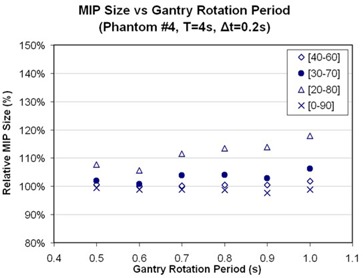
Effect of gantry rotation period on the delineation of phantom 4 (radius: 2.86 cm), shown as a function of various maximum‐intensity pixel (MIP) combination phase windows. The last window (0% – 90%) represents the full range of respiratory motion.

Fig. 7 shows the pixel subtraction images of MIP images constructed by the phase images of static and mobile phantoms acquired under various gantry rotation speeds. A plot of the relative differences in the images of the phantoms measured from the subtraction images is also provided in Fig. 8 for one of the phantoms. The gantry rotation speed controls the temporal length of the projection data in the reconstructed images, and images acquired with faster gantry rotations will be closer to a perfect snapshot in time and therefore closer to the static images used for comparisons. This effect is demonstrated very clearly in Fig. 8, in which target delineation errors were decreased in comparison with the static images as the rotation speed of the gantry increased. For a respiration motion period of 4 s, an image with a 1‐s temporal reconstruction length spans a phase range of ±12.5%. On the other hand, as the rotation speed is doubled for the same motion, the reconstructed images span a phase range of ±6.25%. The dependence on gantry rotation speed also showed a correlation with the size of the MIP phase window selected about the end of exhalation phase ([40% – 60%], [30% – 70%], and [20% – 80%]). The effect of gantry rotation speed was less apparent for smaller window sizes because the narrow phase window selections incorporated less residual motion. In addition, the phantom sizes in the MIP images corresponding to the full range of motion (that is, [0% – 90%]) showed no notable change with respect to their static counterparts and showed no dependence on gantry rotation speed.

## IV. CONCLUSIONS

We studied the dependence of target delineation accuracy on the temporal resolution factors effecting 4DCT acquisitions. The temporal spacing parameter was found to control the temporal accuracy of the reconstructed phase images. Choosing finer temporal spacing below a given threshold was observed to have no change on image integrity and to unnecessarily increase the number of images created in the 4DCT study. On the other hand, very large temporal spacing values resulted in under‐sampling the range of phase images, causing an increase in phase errors for images assigned to a particular phase bin. Therefore, the temporal spacing between the reconstructed images requires that users carefully adjust their selection based on the period of the motion and the number of phase bins created. We recommend that temporal spacing be optimized such that phase errors in any phase bin be smaller than the half‐width of the phase bin.

In addition, an improvement in delineation accuracy with faster gantry speeds was also observed because of acquisition of the projection data within a shorter time period. However, this correlation varied when different ranges of motion were investigated. Size of the phantoms in MIP images constructed from full range of motion phases showed no change with respect to the rotation speed because an increased rotation period or reconstruction interval incurred no extra residual motion. However, large phase windows selected around the end of exhalation were prone to more residual motion and were observed to benefit the most from faster gantry speeds. The effect of rotation speed was less significant when shorter ranges of phase images were analyzed. In no case did faster gantry rotation speeds involve a penalty in terms of image quality. However, in some cases, increasing the tube current with increased rotation speeds required more frequent tube cooling.

## ACKNOWLEDGMENTS

The authors thank John A. Antolak, Sid Whitlock, and Patrick Caskey for the development and construction of the motion platform. This research was partially funded by a research agreement with GE Healthcare Technologies (Waukesha, WI).
